# The Urban–Rural Heterogeneous Effect of Family SES on Achievement: The Mediating Role of Culture

**DOI:** 10.3390/bs14020084

**Published:** 2024-01-24

**Authors:** Ningning Wang

**Affiliations:** School of Sociology, Huazhong University of Science and Technology, Wuhan 430074, China; elaine8811@hotmail.com

**Keywords:** family SES, achievement, culture, heterogeneous effect, urban–rural educational equalization

## Abstract

Previous studies have fully discussed the relationship between family socioeconomic status (SES) and achievement, but few of them regarded urban–rural differences as the moderating factor, which is an essential issue in urban–rural educational equalization in terms of educational outcomes. This study discusses the urban–rural heterogeneous effect of family SES on achievement and manifests the mediating role of education-related cultural factors. Based on the China Education Panel Survey data of 18,672 junior high school students, this study found the following: (1) family SES had a weaker positive effect among rural children than among urban children; (2) the urban–rural heterogeneous effect could be mediated by education-related cultural factors, i.e., learning environments and beliefs; and (3) in this regard, contemporary China is experiencing cultural reproduction rather than a cultural mobility mode. In Conclusion, although the urban–rural achievement gap will be maintained or even expanded in China, improving rural children’s learning environments and beliefs opens up the possibility of alleviating disadvantages in achievement resulting from family socioeconomic disadvantages. Therefore, narrowing down the urban–rural achievement gap from a cultural perspective provides policy implications for educational development in rural areas.

## 1. Introduction

Urban–rural inequality in educational outcomes is a classic and vital issue worldwide, and urban–rural education gaps are especially huge in developing countries, such as Russia [1], Ghana [2], and Vietnam [3]. In contemporary China, despite the educational expansion that has occurred in the last two decades, effectively increasing the educational opportunities for rural children, the urban–rural gap has not narrowed, especially in terms of educational outcomes, and the concern of urban–rural educational inequality is still a crucial social problem [4,5,6].

Family socioeconomic status (SES) is considered an essential factor in explaining educational inequality in the existing literature from both Eastern and Western countries. Scholars agree that there is a correlation between family SES and various educational outcome variables [7,8,9,10], and this family SES effect is stable across geographic and temporal borders [11]. According to the literature, parental advantages can be transmitted to children through two paths. First, parental advantages can directly enhance children’s achievements by the provision of high-quality educational resources [12] (p. 29), including both in- and out-of-school educational resources. In-school resources focus on the school environment, teaching facilities, and faculty strength within the school [13,14,15], whereas out-of-school resources emphasize summer programs, extracurricular activities, private tutoring, and shadow education [16,17,18,19]. Second, family SES indirectly affects children’s achievements through specific cultural factors, including education-related attitudes, educational expectations, family cultural capital, parent–child interactions, and family tutoring [13,20,21,22], all of which are important mediating variables connecting family SES and children’s achievements.

All the aforementioned studies assumed that the influence of family SES on achievement does not vary among different groups of children when explaining educational inequality; however, this assumption is debatable. Transferring family SES advantages to children’s achievements requires specific resources, culture, and environments which are usually nested in social structure. Children in different social structural positions may face drastic distinctions in their educational environments and resources. Therefore, the influence of family SES on children’s achievements may differ in groups with different structural attributes. In Western literature, researchers have proposed that race is an essential structural factor that moderates the association between family SES and achievement. In explaining why Asian Americans academically outperform White people despite their socioeconomic disadvantages, scholars have stated that family SES has a more significant impact on their achievements, whereas Asian Americans rely on positive educational attitudes and high educational expectations to promote their achievements [23,24]. Household registration, which is unique in China, is another significant structural factor within the Chinese context. The household registration system is a fundamental system in China for population regulation based on place of residence. Household registration can be transferred from generation to generation, which means children’s household registrations will follow one of their parents’ household registrations, regardless of their actual living areas. Location and type are the two most crucial attributes of the household registration system. Location distinguishes the place people live according to Chinese administration planning, and type divides people into agricultural and non-agricultural household registration according to rural/urban types of residence. In this study, we focus on the different types of household registration. We regard people holding agricultural household registration as rural residents, and people holding non-agricultural household registration as urban residents. The household registration system, established by the urban–rural binary system, divides the population into urban and rural residents. People holding different household registrations may have significant differences not only in terms of geographic living area, but also in resources, environment, and culture. As a result, such a structural divide may lead to different processes of transforming family SES into achievement among urban and rural children. Such a conclusion is supported by empirical studies with similar results. For example, a Chinese study discovered the distinct impact of family SES on achievement by analyzing different pathways of children’s school learning. The study showed that family SES had a significantly more positive impact on achievement among urban children than among rural children. In contrast, rural children’s achievements are more closely related to their learning behaviors [25]. The moderating role of household registration is an essential issue for urban–rural educational equalization because it shows different patterns of family SES and achievement associations between rural and urban areas, and this may lead to different strategies for improving children’s achievements, especially for resource allocation and policy orientation in rural areas. However, in the previous literature, few studies systematically analyzed the relationship between household registration, family SES, and achievement. Given this literature gap, this study aims to examine the moderating role of household registration in the relationship between family SES and achievement by asking the research question: Does family SES have a heterogeneous effect on achievement across urban and rural children?

In the context of Chinese household registration system, compared with children from rural areas, urban families are more likely to possess better family SES, which is due to unequal economic development between urban and rural areas in China, and such advantages of urban families can be transformed into better achievements for urban children in both direct and indirect ways, as mentioned before. In addition, since resources and culture are key factors in the processes of converting family SES into achievement [21,26,27,28] and there are limited educational resources and a negative culture around education for rural children, they are more likely to have lower efficiency in converting family SES into achievement than their urban counterparts. Based on the above analysis, Hypotheses 1 and 2 are proposed.

**Hypothesis** **1.** *Urban children generally have better achievements than rural children because of their higher family SES*.

**Hypothesis** **2.** *Family SES has a heterogeneous effect on achievement among urban and rural children. Compared with urban children, family SES has a weaker positive effect on achievement among rural children*.

In addition to the patterns of “Family SES-achievement” among different groups, the internal mechanisms and implications are studied as well. Studies from Western countries demonstrated that the heterogenous effect of family SES on achievement in terms of race is through culture. Researchers found that Asian American children tend to perform better academically than White children [15]. Why can Asian Americans achieve academic success despite their relatively low family SES? Researchers suggest that, compared to White families, Asian American families are more serious about education and act upon a stronger belief that education facilitates upward mobility. Owing to the unique Asian education culture, Asian Americans are more likely to achieve positive educational outcomes, even if they have disadvantages in family SES [24,29]. Furthermore, cultural factors moderate the relationship between family SES and educational outcomes. Compared with White people, family SES has a minor influence on achievement and cultural factors among Asian Americans, who are more influenced by Asian culture. This conclusion suggests a heterogeneous impact of family SES on educational outcomes based on race, in which culture plays an essential role [23].

Chinese scholars also noticed culture as a mediating mechanism between family background and achievement by focusing on cultural factors, including educational expectations, cultural capital, cultural environment, and cultural beliefs [30,31,32,33,34]. An empirical study showed that the advantages of parental education can be transmitted to children through cultural reproduction. Educational expectations, cultural capital, and human capital are the three main mechanisms in this process [21]. Another study suggested that the effect of cultural capital on achievement varies across social classes and schools. Compared to children from low-SES families and lower-quality schools, cultural capital has a more significant promoting effect on achievement among children from high-SES families and higher-quality schools; the author calls this “dual reproduction” [22].

The above literature revealed the analytical framework of the racial moderating effect by emphasizing the mediating role of the cultural differences of race within the Western context. Chinese studies also demonstrated the cultural influence on educational inequality. However, the existing studies provide unclear information on the combination of the cultural mediating effect and household registration moderating effect. This study aims to explore the internal mechanism of urban–rural heterogeneous effects of family SES on children’s achievements and will try to analyze the mediating role of culture.

Although different moderating structural variables may lead to different mechanisms, the analytical frameworks of race offer significant implications for comprehending urban–rural educational inequality in China. Traditional Confucianism culture influences both urban and rural Chinese families, but cultural disparities still exist between urban and rural areas in terms of education. Middle-class families in cities with higher family SES tend to share an elite culture around education to prevent downward mobility for the next generation. They usually aim for higher education and regard educational success as one of the most valuable events in their children’s lives [35]. In contrast, rural educational culture is more practical. Rural families with relatively high family SES may possess passive values on education due to the low rate of return from education in rural areas [36], and their children may form negative learning beliefs, attitudes, and behaviors, and enter schools with disadvantaged learning environments and atmospheres. Therefore, the advantages of family SES can be converted differently into positive education-related cultural factors for urban and rural families, leading to urban–rural achievement gaps as a consequence. In this sense, education-related cultural factors mediate the heterogeneous effects of family SES on achievement. The education-related cultural factors in this study refer to the most essential learning attributes influenced by culture, as mentioned before, i.e., learning environments and beliefs. Based on the above analysis, Hypotheses 3 and 4 are proposed.

**Hypothesis** **3.** *Family SES has a heterogeneous effect on education-related cultural factors among urban and rural children. Compared with urban children, family SES has a weaker positive effect on education-related cultural factors among rural children*.

**Hypothesis** **4.** *Education-related cultural factors mediate the urban–rural heterogeneous effect of family SES on achievement*.

By exploring the different urban and rural family SES achievement patterns, and the internal mechanism of culture, this study aspires to discuss a more theoretical issue regarding education in Chinese society, that is, will urban–rural educational inequality be reduced, maintained, or expanded?

There are two competing theories on educational inequality in Western literature: cultural mobility and cultural reproduction theory. According to industrialization theory, with widespread industrialization and a more specialized labor division, parental resources and advantages from family SES cannot be directly transmitted to children. Instead, children gain these advantages through competition in education and through careers. Due to the continuous expansion of education and the enhancement of educational opportunities, the impact of ascribed and structural factors brought about by family SES on educational outcomes will gradually decrease, whereas the influence of achieved factors, such as effort, attitude, and belief, will increase. In this sense, education will reduce class inequality [37]. Empirical studies also showed that the achievement gap between high- and low-social-class students grows faster during vacation periods than during school hours [38,39], which indicates that children of lower SES families can accomplish upward mobility through the education system by actively acquiring the advantages of prestigious status cultures from high-status socioeconomic families. This is called the cultural mobility model [40]. Another opinion suggests that education may maintain or even expand social class inequality; the most representative theory is the cultural reproduction theory. This theory proposes that social and educational inequality is not at the individual level, but is embedded within social structure. Cultural capital can be transmitted across generations through an educational system within a structure, resulting in social reproduction. Cultural capital refers to the cultural factors embedded in structures and institutions and includes three forms: disposition of the mind and body, cultural goods, and an institutionalized state [41] (p. 235). Families in higher social classes provide their children with prestigious cultural resources that can be transformed into positive educational outcomes and higher occupational status through the education system. Thus, children can maintain the social status of their previous generations and achieve cultural reproduction.

In the Chinese context, studies consistently show that the cultural reproduction model is more suitable for contemporary China in terms of social inequality of class [21,22], but few studies test the two competing theories in terms of urban–rural inequality. This study will contribute to discussing the role of the education system in contemporary China and how Western inequality theories apply in China in terms of urban–rural educational inequality. If Hypothesis 1 is supported, it means that the family SES advantages among urban children can be directly transferred into their achievement advantages. If Hypotheses 2–4 are supported, it means that the family SES advantages among urban children can be indirectly transferred into achievement advantages through education-related cultural variables, and such transfer is more effective for urban children. The moderating role of household registration and the mediating role of culture are tested. Therefore, urban–rural achievement gaps will expand due to existing gaps in family SES between urban and rural children, and contemporary China will experience a cultural reproduction mode rather than a cultural mobility mode.

**Figure 1:**
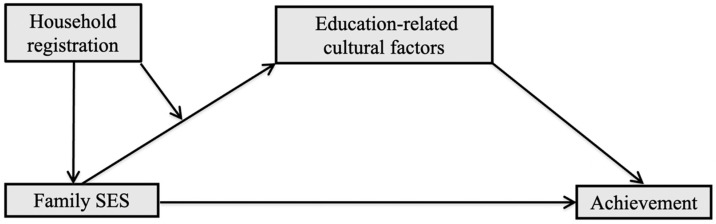
The theoretical framework.

In summary, this study attempts to answer the following research questions: (1) Does household registration modify the relationship between family SES and achievement, and how? (2) Does culture play a mediating role in such a heterogeneous effect in terms of household registration? (3) Which theoretical model, cultural reproduction or cultural mobility mode, is more applicable to contemporary China? Based on the literature, this study hypothesizes that rural children possess not only lower family SES but also weaker associations between family SES and achievement, and as a result rural children will have worse achievements than urban children. We also hypothesize that education-related cultural factors play a mediating role in such a relationship. That is to say, family SES has a weaker positive effect on education-related cultural factors among rural children than their urban counterparts, and this will lead to an urban–rural achievement gap. The relationships of different variables are shown in the theoretical framework (Figure 1). To test the hypotheses, this study plans to use the China Education Panel Survey (CEPS) data of 18,672 junior high school students, including 10,126 rural students and 8546 urban students. Random-effects models and OLS regression with clustered standard errors will be selected in the data analysis due to the research questions and data structure. Furthermore, this study offers policy implications for policymakers concerned with rural educational development and urban–rural equalization in educational outcomes.

This study contributes to the literature in three ways. First, although the existing literature has extensively researched the effect of family SES on children’s educational outcomes, few researchers have noticed that family SES effect varies between urban and rural children. This study enriches the literature on the effect of family SES by examining how family SES influences children’s achievements differently with different household registrations, which helps disentangle a more precise relationship between family SES and educational outcomes. Second, there are a large number of studies discussing the mediating role of cultural factors in educational inequality, but less attention has been paid to the linkage between the moderating role of household registration and the mediating role of culture. This study enriches the literature on the mediating role of cultural variables in urban–rural educational inequality. Third, this study further verifies the applicability of cultural reproduction and mobility models within the Chinese context, enriching the literature on the validation of classical educational inequality theories and providing data support for understanding the patterns and mechanisms of education inequality in contemporary China.

## 2. Materials and Methods

### 2.1. Method

This study used a quantitative method to conduct the analysis. The data were obtained from the China Education Panel Survey (CEPS) of 2013–2014. The participants in the CEPS survey were junior high school students, in addition to parents, teachers, and school leaders. Since this study aims to compare different patterns of family SES on children’s achievements between urban and rural areas, two subsamples were included in this study: children with urban household registration and those with rural household registration. The total sample size was 18,672, including 10,126 sample rural students and 8546 sample urban students. In addition, we also matched students’ family and school information from the parent, home teacher, and school administrator questionnaires with their individual information, which made it possible to test our hypotheses between the dependent variables of children’s achievements, independent variables of family SES and household registration, and mediating variables of education-related cultural variables.

### 2.2. Data Collection

The CEPS (survey website: http://ceps.ruc.edu.cn/English/Overview/Overview.htm, accessed on 20 January 2024; email address: ceps@nsrcruc.org; mailing address: China Education Panel Survey, National Survey Research Center, Renmin University of China, Haidian, Beijing, China, 100872) is a critical routine survey project of the National Survey Research Center (NSRC) at Renmin University of China. The CEPS survey was a large-scale, nationally representative, longitudinal survey that focused on the linkages between adolescents’ educational outcomes and multiple factors related to families, schools, communities, and social structures. The CEPS applied a stratified sampling design of probability proportional to size (PPS), randomly selecting students in 438 classrooms of 112 schools from 28 county-level units in mainland China. The sampling procedure involved 4 stages. First, select 28 primary sample units (PSUs) with PPS. Second, select 4 schools from each sample county/district with PPS. Third, select 2 classrooms, respectively, in each grade and each sample school. Fourth, include all students attending school on the survey date in the selected classrooms. The CEPS administered not only student questionnaires, but also questionnaires to sample parents, homeroom teachers, main subject teachers who were not homeroom teachers, and school administrators. As a result, the CEPS data are in a hierarchical structure at individual, class, and school levels. In addition, CEPS also conducted standardized cognitive ability tests for students in each grade, respectively.

### 2.3. Variables

#### 2.3.1. Dependent Variables

The core dependent variable in this study was children’s cognitive and academic achievement. In the first part of the analysis, which concerned the heterogeneous effect of family SES, this study selected the cognitive ability test score to represent cognitive achievement, and exam scores of three major subjects (Chinese, Mathematics, and English) from the survey data as indicators of students’ academic achievements. The cognitive ability test is a standardized test developed for students taking the CEPS survey that aims to test their logical thinking and problem-solving abilities. The test involves 3 dimensions and 11 concepts, including (1) language (analogy of phrases and linguistic reasoning); (2) graphics and space (analysis of graphic patterns, origami questions, and application of geometry); and (3) calculation and logic (mathematical applications, custom calculation rules, sequence application, abstract pattern analysis, probability, and reverse thinking of numbers). As the total scores of the cognitive ability test were not the same for participants from different grades, this study converted the cognitive ability test scores into percentages ranging from 1 to 100 within the same grade, representing the student’s ranking in the cognitive ability test. In addition, this study also standardized the exam scores of three major subjects with a mean of 70 and a standard deviation of 10 within schools and grades because of the varied difficulties and total scores across different schools. The standardization made the score comparable across different schools; however, it has limitations as well. It is impossible to standardize the difficulty of exams in different schools, and this may lead to deviations between the standardized scores and students’ real achievements and abilities. Students with similar abilities may have different standardized scores in different schools.

In the second part of the analysis, which focuses on the cultural mediation mechanism of the urban–rural heterogeneous effect, this study used principal component analysis (PCA) to construct the achievement score, which consists of cognitive ability test score and the standardized scores of three major subjects for simplicity and convenience in the data analysis. The achievement score was standardized with a mean of 70 and standard deviation of 10 across schools and grades. Based on the Kaiser–Meyer–Olkin (KMO) test and Bartlett test, it is appropriate to use PCA for the achievement score. This study chose 2 principal components to construct the comprehensive score of achievement, and the cumulative proportion was 0.824. More detailed information about PCA is shown in Appendix A. 

#### 2.3.2. Independent Variables

The core independent variables were students’ household registration, family SES, and the interaction variable between children’s household registration and family SES. Household registration is a binary variable (0 = rural; 1 = urban). Family SES is a comprehensive variable recognized as the most explanatory variable to represent family background. The measurement indicators of family SES typically include family income, parents’ educational level, and parents’ occupational status [42,43]. Based on the indicators in the literature, this study used parents’ educational level, occupational status, and self-reported family economic conditions as the leading indicators to measure family SES. These indicators were integrated and processed using PCA to create a non-negative continuous variable of family SES ranging from 0 to 7. Based on the Kaiser–Meyer–Olkin (KMO) test and Bartlett test, it is appropriate to use PCA for family SES. This study chose 3 principal components to construct the family SES variable, and the cumulative proportion was 0.850. More detailed information about PCA is shown in Appendix B. A higher value for this variable indicates higher family SES. The interaction term between household registration and family SES examined whether the family SES effect varied between urban and rural children. It should be noted that, owing to data limitations, this study uses self-reported family economic conditions as a proxy for family income, which may result in a loss of accuracy and objectivity in the measurement. However, since subjective and objective economic statuses are closely related [44], self-reported economic status can still provide strong explanatory power in measuring family SES.

#### 2.3.3. Mediating Variables

Social psychological studies suggest that culture may shape children’s mindsets, and thus their achievements [45]. According to the literature, learning environments and learning beliefs are two crucial cultural variables associated with children’s achievements [22,23,30,31,32]. Learning environments and beliefs have significant differences in urban and rural cultures. Since rural society shares a more practical culture, rural families regard monetary returns as a more important factor than occupational reputation in the future. Therefore, fewer rural children aim to go to college than urban children, and the overall learning environment and educational beliefs are more negative and passive in rural areas than in urban areas.

We use four variables to measure the cultural mediating effect. The educational expectations of the class represent students’ learning environments. Learning beliefs in this study include three aspects: children’s views on the importance of education, children’s opinions on hard work, and children’s study attitudes. The operationalization of the four variables is as follows. The educational expectations of the class variable were calculated by summing the years of education expectations of each student in the class and then averaging them. Students’ expected years of schooling were converted from their educational expectations: 9 = junior high school, 12 = senior high school, 16 = bachelor’s degree, 19 = master’s degree, 22 = doctor’s degree. Higher scores represent a better study environment. The importance of the education variable corresponds to the following questions in the questionnaire: “Regarding the major subjects, do you agree with the following statements: (1) Chinese is helpful for my future, (2) Mathematics is helpful for my future, and (3) English is helpful for my future?” We constructed the importance of education by summing the scores for the above three questions (1 = strongly disagree, 2 = somewhat disagree, 3 = somewhat agree, 4 = strongly agree). Higher scores represent better perceptions of the importance of education. The hardworking variable corresponds to the following questions: “Do you agree with the following descriptions of yourself: (1) Even if I feel a little uncomfortable or have other reasons to stay at home, I will still try to go to school; (2) Even if it is a subject I do not like, I will still do my best; (3) Even if the homework takes a long time to complete, I will still try my best to do it?” We constructed a hardworking variable by summing the scores of the above three statements (1 = strongly disagree, 2 = somewhat disagree, 3 = somewhat agree, 4 = strongly agree). A higher score represents more hard work in studying. The study attitude variable was measured by the parent-rated study attitude of the child from 1 to 5; a higher score represents a more positive study attitude.

#### 2.3.4. Control Variables

Since students’ achievements are highly related to school attributes, school structural variables were added as control variables in this study, including students’ average budget expenditure (RMB), hardware facilities, student–teacher ratio, proportion of teachers with bachelor’s degrees, and proportion of senior teachers. The regional variables included regional location (1 = eastern region, 2 = central region, 3 = western region) and regional type (1 = municipality, 2 = provincial capital, 3 = prefecture-level city, 4 = county-level city). Moreover, students’ achievements will be influenced by children’s demographic variables and regional variables. As a result, these variables were added as control variables in the models as well. Children’s demographic variables included gender (0 = female, 1 = male), grade (0 = grade 7, 1 = grade 9), siblings (0 = does not have siblings, 1 = has siblings), and the regional educational level of parents, which is the average educational level of parents in a particular region. Table 1 shows the descriptive statistics for all the variables.

### 2.4. Data Analysis

The data analysis was divided into two parts. In the first part, we aimed to investigate whether there is an urban–rural heterogeneous effect of family SES on achievement. Owing to the clear hierarchical structure of the data used in this study, with individual-, class-, and school-level data nested, multilevel models were employed. We selected random-effects models at the class level to investigate whether there was an urban–rural heterogeneous effect of family SES on achievement. The model was divided into two levels: the first level was individual, and the second level was class.

In the second part, as education-related cultural variables are related to school selection, we selected OLS regression with clustered standard errors at the school level to investigate whether cultural factors can explain the urban–rural heterogeneous effect of family SES on achievement. The data analysis in this section was achieved through the following two steps: (1) examining the urban–rural heterogeneous effect of family SES on education-related cultural variables, and (2) examining the extent to which these education-related cultural variables can explain the urban–rural heterogeneous effect of family SES on achievement.

## 3. Results

### 3.1. The Urban–Rural Heterogeneous Effect of Family SES on Achievement

In this part of the analysis, descriptive statistics were first used to show the distribution and changes in achievement between rural and urban children from different socioeconomic families. Subsequently, a random-effects model at the class level was used to test whether the distribution and trends shown in the descriptive statistics were statistically significant at the 95% bias-corrected confidence interval (CI).

**Figure 2:**
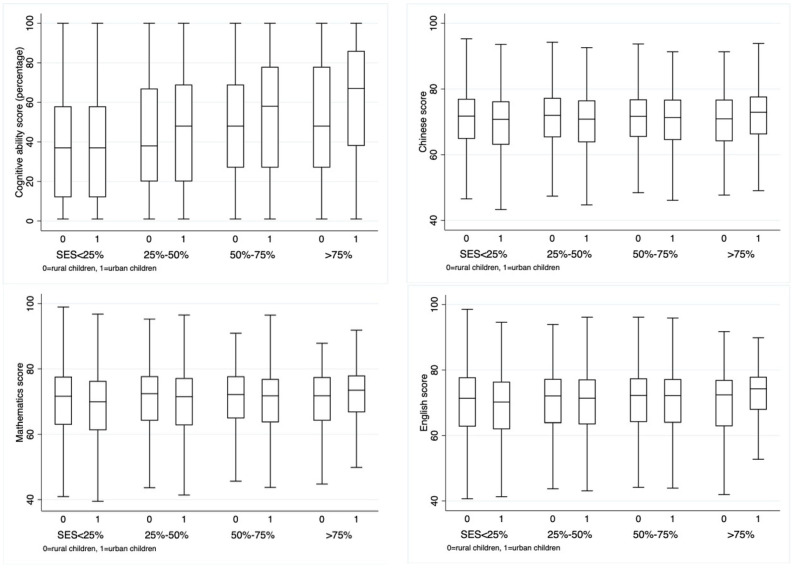
The urban–rural differences in achievement in family SES quartile.

Figure 2 shows the urban–rural differences in achievement with different family SES levels, which suggests that “family SES-Achievement” patterns differ between rural and urban areas. For families with socioeconomic status in the bottom 25%, there were few differences in cognitive ability and English scores between urban and rural children, and urban children even performed worse than their rural counterparts in Chinese and Mathematics. Meanwhile, as the family SES quartile increased, the values of each achievement indicator increased correspondingly, indicating a positive effect of family SES on children’s achievements in both urban and rural areas. However, the urban–rural gap in achievement widened at the same time. As for families with socioeconomic status in the top 25%, urban children had significantly higher scores in cognitive ability, Chinese, Mathematics, and English than rural children.

To further investigate this phenomenon, we used a random-effects model at the class level to test the heterogeneous effect of family SES on achievement (Table 2). The result showed a weaker correlation between family SES and achievement among rural children. Models 1–8 examined four dependent variables representing achievement: cognitive ability, Chinese, Mathematics, and English scores. According to Table 2, family SES had a positive and significant effect on cognitive ability (coefficient = 3.244, *p* < 0.001), Chinese score (coefficient = 0.973, *p* < 0.001), Mathematics score (coefficient = 1.004, *p* < 0.001), and English score (coefficient = 1.174, *p* < 0.001). Moreover, the interaction term between household registration and family SES was positive and significant for the cognitive ability score (coefficient = 1.839, *p* < 0.01), Chinese score (coefficient = 0.477, *p* < 0.05), Mathematics score (coefficient = 0.760, *p* < 0.01), and English score (coefficient = 1.260, *p* < 0.001), which means that the positive family SES effect is stronger for urban children, and there is a weaker correlation between family SES and achievement among rural children. Since there are different categories of rural children, we further examined whether there is a heterogeneous effect of family SES on educational achievement between migrant children (rural children who migrate to urban areas with their parents) and local rural children. However, the results show that the heterogeneous effect of family SES on educational achievement between migrant children and local rural children is not significant.

**Figure 3:**
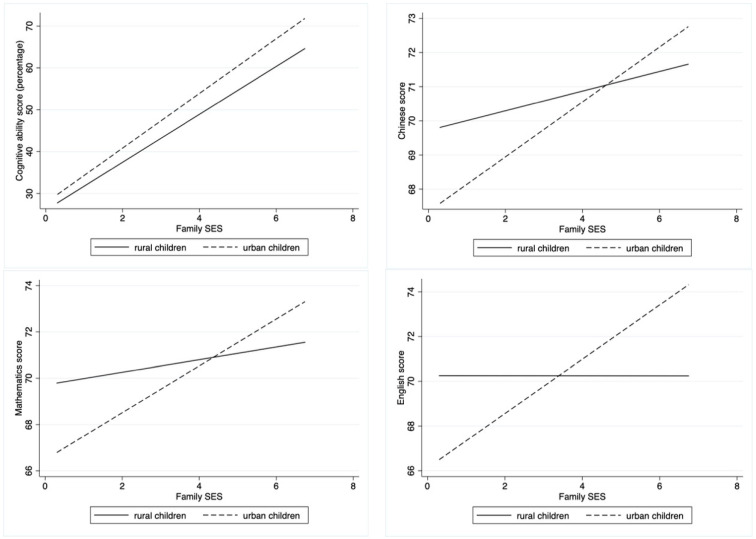
Urban–rural heterogeneous effect of family SES on cognitive ability, Chinese, Mathematics, and English scores.

To observe this phenomenon intuitively, the analysis results are shown graphically for the relationships between family SES and the four dependent variables representing achievement (Figure 3). The slopes for both urban and rural children were positive; however, the slope for urban children was higher than that for rural children for all four dependent variables. This result indicates that family SES positively promotes both urban and rural children’s achievements and has a heterogeneous effect on urban and rural children. Family SES has a tremendous positive promoting effect on urban children’s achievements, and rural children are less influenced by family SES.

Based on the above analysis, the urban–rural difference in achievement is not only caused by the urban–rural gap in family SES, but also by the interactive effect of household registration and family SES. Urban children generally have a higher family SES than rural children, and thus have better achievements. Hypothesis 1 is supported. However, the positive effect of family SES on achievement was less significant among rural children than among urban children. Hypothesis 2 is supported.

### 3.2. The Mediating Role of Education-Related Cultural Factors

The analysis in the previous section revealed the urban–rural heterogeneous effect of family SES on achievement. However, how can we interpret this urban–rural heterogeneous effect? This section attempts to explain the urban–rural heterogeneous effect of family SES from a cultural perspective by adding education-related cultural variables to the models.

Table 3 shows the effect of urban–rural differences in family SES on learning environments and beliefs. Model 9–12 examines whether urban–rural heterogeneity exists in the effect of family SES on each education-related cultural variable. The results show that the interaction term between household registration and family SES is significant and positive for the educational expectations of class (coefficient = 0.205, *p* < 0.01), importance of education (coefficient = 0.205, *p* < 0.01), hard work (coefficient = 0.192, *p* < 0.01), and study attitude (coefficient = 0.129, *p* < 0.001). This result indicates that urban–rural heterogeneity exists in the family SES effect on learning environments and beliefs. Compared with urban children, the positive impact of family SES on education-related cultural variables is less substantial for rural children. Thus, Hypothesis 3 is supported.

The above analysis confirms the urban–rural heterogeneous effect of family SES on education-related cultural variables. Furthermore, we investigated whether these education-related cultural variables accounted for the urban–rural heterogeneous effect of family SES on achievement. For simplicity, models 13–18 in Table 4 use the comprehensive achievement score as the dependent variable.

According to Table 4, Model 13 is the base model that adds only household registration, family SES, and the interaction term. The interaction term in the base model is positive and significant (coefficient = 1.063; *p* < 0.01). In Models 14–17, each education-related cultural variable was added to the base model, respectively. The results show that the educational expectation of the class (coefficient = 1.093, *p* < 0.001), importance of education (coefficient = 0.798, *p* < 0.001), hard work (coefficient = 0.707, *p* < 0.001), and study attitude (coefficient = 3.652, *p* < 0.001) are all positive and significant. At the same time, the coefficient of the interaction term decreased in Model 14 (from 1.063 to 0.855), Model 15 (from 1.063 to 0.930), Model 16 (from 1.063 to 0.933), and Model 17 (from 1.063 to 0.578), when compared with the base model (Model 13). These results indicate that each education-related cultural variable affects achievement and can partially explain the heterogeneous effects of family SES on achievement. In Model 18, all education-related cultural variables were added to the base model at the same time. After controlling for all the education-related cultural variables, the significance of the interaction term disappeared, indicating that education-related cultural variables can fully explain the effect of the urban–rural heterogeneity of family SES on achievement. Thus, Hypothesis 4 is supported. We also noticed that the family SES variable was still significant in this model (coefficient = 0.867, *p* < 0.001), indicating that although learning environments and beliefs can explain the heterogeneous effect of family SES, the direct effect of family SES on achievement still exists.

## 4. Discussion

This study indicated different patterns between family SES and achievement in urban and rural areas, and the relationship between family SES and achievement was stronger in urban areas than in rural areas. The findings respond to the first research question, and the findings are consistent with the patterns of the previous study by Wu et al. [22]. In addition, this study also revealed that the urban–rural heterogeneous effect of family SES on achievement is the result of the urban–rural heterogeneous effect of family SES on learning environments and beliefs, which determines that education-related cultural factors are important to academic success. The results answer the second research question. The mediating effect of culture is similar to that found in Western studies [15,23]. Given the giant economic development gap between urban and rural areas, urban children not only have better family SES but can also more effectively transform SES advantages into achievement advantages through learning environments and beliefs, which may encourage them to obtain higher education levels and occupational status in the future. Owing to the above findings, Chinese society remains in a mode of cultural reproduction, and our conclusion confirms previous studies regarding class inequality [21,22], which answers the third research question. “Impoverished families can hardly nurture rich sons” is a popular phrase in China, meaning that children from low SES families can hardly achieve success. Such a phrase reflects the public opinion that deems family SES a strong predictor of academic success. We concede that the family SES gap plays a vital role in urban–rural educational inequality in China and other countries [1,46,47], and with economic development and increasing income in both rural and urban areas, the urban–rural educational gap may further expand in the future due to the pattern of family SES heterogeneous effect shown in this study. The education system continues to play a role in maintaining or expanding the inequality of urban and rural education under the existing household registration and education system.

However, the weaker relationship between achievement and family SES in rural areas suggests that the monetary influence of the family is less important for rural children’s achievements, and their achievements rely more on cultural factors, which is similar to the conclusion of a previous study [25]. As a result, improving rural children’s learning environments and beliefs opens up possibilities of alleviating the disadvantages in cognitive and academic achievement due to lower family SES because these cultural factors are not rigidly associated with family SES. In this sense, learning environments and beliefs play a positive role in breaking intergenerational status replication and suggest the possibility that children from low-SES families may have positive educational outcomes and future career development.

The above discussions yield policy implications from a cultural perspective for promoting urban–rural equalization. First, they promote the culture revitalization program in rural areas, which is one of the five essential aspects of the rural revitalization strategy in China. The rural revitalization strategy was proposed at the 19th CPC National Congress in 2017 in China. The goal of the rural revitalization strategy is to promote the development of rural areas. It includes five dimensions, and culture revitalization is one of the five revitalizations. Central government needs to pay more attention to this cultural aspect by improving the overall educational attitudes and beliefs of children, educators, and families in rural counties. Cultural lag theory points out that in the process of social change, changes in material culture always occur before changes in non-material culture [48]. This theory implies that, although the poverty alleviation program in China has dramatically improved the hardware infrastructure in rural areas, changes in the cultural environment of rural schools and families may be left behind. In contemporary China, many rural parents, and even teachers, still think that education is useless for children’s future development. Such beliefs lead to low expectations, unclear study motivation, and negative study behavior, and harm educational outcomes as a result. In this context, the government needs to put more effort into helping rural educators and families establish positive attitudes and beliefs toward education. Second, education informatization strategies, like massive open online courses (MOOCs) and mobile learning, should be encouraged in rural areas. Education informatization will not only share urban education resources to rural areas, but also promote urban–rural cultural integration. Although there is no level of superiority between urban and rural cultures, urban culture is more conducive to children’s educational outcomes than rural culture. Therefore, actively achieving education-related urban culture will benefit rural children in the process of cultural integration. With improvements in Internet hardware, online education facilitates rural children not only in enhancing educational resources, but in promoting positive urban culture regarding educational beliefs, attitudes, and environments. Through online education, rural children are more likely to be influenced by positive urban culture and further enhance their educational outcomes.

This study has certain limitations. First, the research findings are based on a linear relationship between family SES and achievement; however, a nonlinear relationship is possible. One possibility is that higher family SES promotes children’s achievements more than lower family SES. On average, urban children have higher family SES than rural children, which may result in a more significant family SES effect in urban areas. To further investigate this issue, the author divided the sample into two subsamples based on family SES (high- and low-SES subsamples). In the models of the two subsamples, the interaction term of household registration and family SES was significant for children’s achievements in the high-SES subsample, but insignificant in the low-SES subsample (due to space limitations, these two models are not presented in the article). The results suggest that the impact of family SES on achievement varies among different SES subsamples, and a nonlinear relationship is possible. However, even if there is a nonlinear relationship, the urban–rural heterogeneous effect of family SES on achievement still exists in the subsample with higher SES. Therefore, the results of the present study are still valid. Second, due to data limitations, the measurements of certain variables are not precise enough, which may affect the reliability and validity of the statistical results. It is noted that self-reported economic conditions as a proxy may not reflect family income objectively, and standardized exam scores in Chinese, Math, and English may still have problems in comparison across different schools because of different difficulties. However, subjective and objective economic status are highly correlated [42], and the patterns of family SES effect on exam scores are consistent with family SES effect on the cognitive achievement score. Therefore, the two variables are valid in the statistical analysis.

Future research can be expanded into three aspects. First, further discussion can be conducted on how the heterogeneous effect and nonlinear relationship commonly influence children’s achievements to more accurately clarify the relationship between family SES, household registration, and achievement. Second, this study only examined the mediating effect of learning environments and beliefs; more cultural factors could be included in the framework of family SES heterogeneous models in future studies. Third, this study is based on the background of the unique Chinese binary household registration system, but the issue of urban–rural educational inequality is a worldwide issue. More comparisons across countries can be conducted in the future.

## 5. Conclusions

Does the effect of family SES differ between urban and rural children? Can learning environments and beliefs explain this phenomenon? This study used an empirical analysis of the China Education Panel Survey data of junior high school students. The following conclusions were drawn: First, family SES has a positive impact on cognitive and academic achievement for both urban and rural children; however, this positive effect of family SES has urban–rural heterogeneity. Compared to urban children, family SES has a weaker positive effect on cognitive and academic achievement among rural children. Second, education-related cultural variables, i.e., learning environments and beliefs, are crucial mediating variables that explain the urban–rural heterogeneous effect of family SES on achievement. Compared with urban children, there is a weaker correlation between family SES and education-related cultural variables among rural children, and this will result in the urban–rural heterogeneous effect of SES on achievement. Third, China is currently experiencing a cultural reproduction mode rather than cultural mobility, which means urban–rural educational inequality in China will be maintained, and even expanded, in the future. More effort needs to be conducted to improve rural learning environments and beliefs to buffer the negative effect of family disadvantages in rural areas through the culture revitalization program and educational informatization.

## Figures and Tables

**Table 1:** Descriptive statistics.

Variable Name	Mean	SD	Min	Max	Variable Level
**Dependent variable**					
Cognitive ability score (percentage)	46.72	28.99	1	100	Individual
Chinese score	69.99	10.10	6.16	98.47	Individual
Mathematics score	70.11	9.83	8.42	145.11	Individual
English score	70.11	9.85	11.35	107.82	Individual
Achievement score	70.10	9.94	17.59	100	Individual
**Independent variable**					
Household registration (1 = urban)	0.45	0.50	0	1	Individual
Family SES	3	1.03	0.30	6.75	Individual
**Mediating variable**					
Educational expectation of the class	16.42	1.35	11.72	21.05	Class
Importance of education	9.78	2.04	3	12	Individual
Hardworking	9.93	2.01	3	12	Individual
Study attitude	3.32	0.96	1	5	Individual
**Control variable**					
Gender (1 = male)	0.51	0.50	0	1	Individual
Grade (1 = grade 9)	0.47	0.50	0	1	Individual
Siblings (1 = have siblings)	0.57	0.50	0	1	Individual
Students’ average budget expenditure (RMB)	952.02	678.78	0	3850	School
Hardware facility	20.87	4.12	12	30	School
Student–teacher ratio	11.98	4.63	3.44	33.2	School
Proportion of teachers with bachelor’s degree	0.79	0.23	0.015	1	School
Proportion of senior teachers	0.22	0.15	0	0.73	School
Regional type	2.97	1.13	1	4	Region
Regional location	1.70	0.84	1	3	Region
Regional educational level of parents	9.49	1.44	6.8	12.19	Region

**Table 2:** Model of the urban–rural heterogeneous effect of family SES on achievement (a random-effects model at the class level).

	Model 1	Model 2	Model 3	Model 4	Model 5	Model 6	Model 7	Model 8
	Cognitive Ability		Chinese		Mathematics		English	
Household registration (1 = urban)	0.129	−5.106 *	−5.111	−1.868 *	−0.785 *	−2.951 ***	−0.231	−3.816 ***
Family SES	3.244 ***	2.065 ***	0.973 ***	0.666 **	1.004 ***	0.514 *	1.174 ***	0.362
Household registration * family SES		1.839 **		0.477 *		0.760 **		1.260 ***
Control variable	Have controlled		Have controlled		Have controlled		Have controlled	
Overall R^2^	0.162	0.164	0.098	0.099	0.012	0.013	0.094	0.097
Sample size	7411	7411	7241	7241	7234	7234	7235	7235

Note: (1) Control variables in this model included the child’s gender, having siblings or not, students’ average budget expenditure, hardware facilities, student–teacher ratio, the proportion of teachers with bachelor’s degrees, proportion of senior teachers, regional location, regional type, and regional educational level of parents; (2) * *p* < 0.05; ** *p* < 0.01; *** *p* < 0.001 (two-tailed test).

**Table 3:** Model of the urban–rural heterogeneous effect of family SES on education-related cultural factors (OLS regression with clustered standard errors at the school level).

	Model 9	Model 10	Model 11	Model 12
	Educational Expectation of the Class	Importance of Education	Hardworking	Study Attitude
Household registration (1 = urban)	−0.470 *	−0.625 **	−0.673 **	−0.405 **
Family SES	0.012	−0.047	−0.121 *	0.255
Household registration * family SES	0.205 **	0.205 **	0.192 **	0.129 **
Control variable	Have controlled	Have controlled	Have controlled	Have controlled
R^2^	0.556	0.057	0.057	0.073
Sample size	7411	7374	7178	7174

Note: (1) Control variables in this model included the child’s gender, grade, having siblings or not, students’ average budget expenditure, hardware facilities, student–teacher ratio, the proportion of teachers with bachelor’s degrees, proportion of senior teachers, regional location, regional type, regional educational level of parents; (2) * *p* < 0.05; ** *p* < 0.01 (two-tailed test).

**Table 4:** Model of the mediating effect of education-related cultural factors (OLS regression with clustered standard errors at the school level).

	Model 13	Model 14	Model 15	Model 16	Model 17	Model 18
Household registration (1 = urban)	−3.356 **	−2.929 **	−2.944 **	−2.901 **	−1.849 *	−1.299
Family SES	0.903 ***	0.884 ***	0.929 ***	0.979 ***	0.825 ***	0.867 ***
Household registration * family SES	1.063 **	0.855 *	0.930 **	0.933 **	0.578 *	0.362
Educational expectation of class		1.093 ***				0.704 *
Importance of education			0.798 ***			0.415 ***
Hardworking				0.707 ***		0.305 ***
Study attitude					3.652 ***	3.322 ***
Control variable	Have controlled	Have controlled	Have controlled	Have controlled	Have controlled	Have controlled
R^2^	0.079	0.092	0.105	0.099	0.199	0.220
Sample size	6745	6745	6745	6745	6745	6745

Note: (1) Control variables in this model included the child’s gender, grade, having siblings or not, students’ average budget expenditure, hardware facilities, student–teacher ratio, the proportion of teachers with bachelor degrees, proportion of senior teachers, regional location, regional type, regional educational level of parents; (2) * *p* < 0.05; ** *p* < 0.01; *** *p* < 0.001 (two-tailed test).

## Data Availability

The dataset used in this study is publicly archived, and the link is http://ceps.ruc.edu.cn/English/Overview/Overview.htm, accessed on 20 January 2024.

## Appendix A. PCA for Achievement Score

**Table A1:** Kaiser–Meyer–Olkin (KMO) test and Bartlett test of sphericity.

Method	Index	Result
KMO test	KMO	0.749
Bartlett test	Chi-square	25,422.064
	Degrees of freedom	6
	*p*-value	0.000

**Table A2:** Principal component proportion.

Component	Eigenvalue	Difference	Proportion	Cumulative
Comp1	2.467	1.638	0.617	0.617
Comp2	0.830	0.427	0.207	0.824
Comp3	0.403	0.103	0.101	0.925
Comp4	0.300	-	0.075	1.000

**Table A3:** Eigenvectors.

Variable	Comp1	Comp2	Comp3	Comp4
Cognitive ability score	0.331	0.932	0.141	−0.043
Chinese	0.537	−0.259	0.610	0.521
Math	0.542	−0.060	−0.775	0.318
English	0.555	−0.246	0.082	−0.791

Calculation

Achievement score = (0.617/0.824) Comp1 + (0.207/0.824) Comp2

## Appendix B. PCA for Family SES

**Table A4:** Kaiser–Meyer–Olkin (KMO) test and Bartlett test of sphericity.

Method	Index	Result
KMO test	KMO	0.718
Bartlett test	Chi-square	25,004.961
	Degrees of freedom	10
	*p*-value	0.000

**Table A5:** Principal component proportion.

Component	Eigenvalue	Difference	Proportion	Cumulative
Comp1	2.635	1.767	0.527	0.527
Comp2	0.867	0.117	0.173	0.700
Comp3	0.749	0.296	0.150	0.850
Comp4	0.453	0.158	0.090	0.941
Comp5	0.294	-	0.058	1.000

**Table A6:** Eigenvectors.

Variable	Comp1	Comp2	Comp3	Comp4	Comp5
Mother’s educational level	0.495	0.021	−0.500	−0.322	−0.632
Father’s educational level	0.491	0.012	−0.510	0.364	0.604
Mother’s occupational status	0.467	−0.307	0.410	−0.628	0.351
Father’s occupational status	0.460	−0.286	0.475	0.607	−0.333
Family economic conditions	0.285	0.907	0.308	−0.018	0.020

Calculation

Family SES = (0.527/0.850) Comp1 + (0.173/0.850) Comp2 + (0.150/0.850) Comp3
